# Broadband Two-Photon Absorption Characteristics of Highly Photostable Fluorenyl-Dicyanoethylenylated [60]Fullerene Dyads

**DOI:** 10.3390/molecules21050647

**Published:** 2016-05-14

**Authors:** Seaho Jeon, Min Wang, Wei Ji, Loon-Seng Tan, Thomas Cooper, Long Y. Chiang

**Affiliations:** 1 Department of Chemistry, Institute of Nanoscience and Engineering Technology, University of Massachusetts Lowell, Lowell, MA 01854, USA; baschem@hotmail.com (S.J.); wangmin81@gmail.com (M.W.); 2 Department of Physics, National University of Singapore, Singapore 117542, Singapore; 3 Functional Materials Division, AFRL/RXA, Air Force Research Laboratory, Wright-Patterson Air Force Base, Dayton, OH 45433, USA; loon.tan@us.af.mil (L.-S.T.); thomas.cooper.13@us.af.mil (T.C.)

**Keywords:** C_60_-(light-harvesting antenna) nanostructures, Z-scan measurements, ultrafast two-photon absorption, nonlinear absorption

## Abstract

We synthesized four C_60_-(light-harvesting antenna) dyads C_60_ (>CPAF-C_n_) (*n* = 4, 9, 12, or 18) **1**-C_n_ for the investigation of their broadband nonlinear absorption effect. Since we have previously demonstrated their high function as two-photon absorption (2PA) materials at 1000 nm, a different 2PA wavelength of 780 nm was applied in the study. The combined data taken at two different wavelength ranges substantiated the broadband characteristics of **1**-C_n_. We proposed that the observed broadband absorptions may be attributed by a partial π-conjugation between the C_60_ > cage and CPAF-C_n_ moieties, via endinitrile tautomeric resonance, giving a resonance state with enhanced molecular conjugation. This transient state could increase its 2PA and excited-state absorption at 800 nm. In addition, a trend of concentration-dependent 2PA cross-section (σ_2_ ) and excited-state absorption magnitude was detected showing a higher σ value at a lower concentration that was correlated to increasing molecular separation with less aggregation for dyads C_60_(>CPAF-C_18_) and C_60_(>CPAF-C_9_), as better 2PA and excited-state absorbers.

## 1. Introduction

Nonlinear optical materials have numerous applications, including photodynamic therapy, nonlinear photonics, 3D optical data storage, frequency upconverted lasing, and fluorescence imaging [1,2,3,4,5,6,7,8,9,10]. These materials require large two-photon absorption (2PA) cross-section (σ_2_) of nonlinear absorbers [11,12,13]. Carbon-based materials, e.g., fullerenes, nanocarbons (NC), and carbon nanotubes (CNT), are suitable as the base substrate for fabricating potential 2PA absorbers. Among them, unlike NC and CNT, fullerenes are highly versatile toward various chemical functionalization reactions with good efficiency to form soluble derivatives that facilitate the material engineering processing and coating fabrication.

We have recently reported dual NIR nonlinear optical absorption activities of branched triad C_60_(>DPAF-C_18_)(>CPAF-C_2M_) and tetrad C_60_(>DPAF-C_18_)(>CPAF-C_2M_)_2_ nanostructures, each consisting of hybrid light-harvesting antenna DPAF-C_n_ and CPAF-C_n_ moieties [14]. These two types of chromophores were designed to provide linear optical absorption [or one-photon absorption (1PA)] at 400 and 500 nm, respectively, which corresponds to 2PA excitation at 800 and 1000 nm, respectively. By increasing the number of CPAF-C_n_ to two in the corresponding tetrad, the sum of extinction coefficients of overlapped DPAF–CPAF absorption bands at 400–550 nm led to a spectrum profile with a nearly flat band over this wavelength range indicating broadband characteristics. Ultrafast femtosecond (fs) 2PA cross-section values of 266 and 995–1100 GM (125 fs, at 5.0 × 10^−3^ M in toluene) were reported for the hybrid tetrad at 780- and 980-nm irradiation, respectively [15]. The σ_2_ value was higher than that (30 GM in CS_2_, 1.0 × 10^−2^ M) of the dyad C_60_(>DPAF-C_9_) at 780-nm excitation. The enhancement was correlated to efficient intramolecular Förster resonance energy-transfer events going from a high-energy DPAF-C_n_ antenna unit to low-energy CPAF-C_n_ antenna units occurring in a cascade fashion at the C_60_ > cage surface.

**Figure 1:**
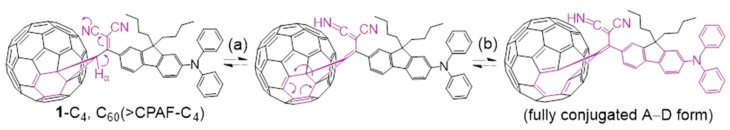
(**a**) Endinitrile tautomeric resonances at the bridging C_61_H_α_‒C[=C(CN)_2_] structure of a C_60_-CPAF conjugate compound **1**-C_4_ and (**b**) fullerenyl seven-membered ring expansion involving C_61_, leading to the formation of a fully-conjugated form of C_60_> acceptor (**A**) and CPAF donor (**D**), as marked in purple. This resulted in an extended **A**–**D** conjugation length and absorption wavelengths, marked in burgundy red in color.

Further investigation on the molecular structure of 7-(1,2-dihydro-1,2-methano [60]fullerene-61-{1,1-dicyanoethylenyl})-9,9-dialkyl-2-diphenylaminofluorene C_60_ (>CPAF-C_n_) **1**-C_n_ led us to propose that a partial π-conjugation between the C_60_> cage and CPAF-C_n_ moieties, via endinitrile tautomeric resonances or isomerization (Figure 1), may merge the 2PA wavelength of fullerene cage at 600–700 nm together with that of CPAF-C_n_ (900–1100 nm) at the photoexcited state. This may simulate the 2PA of DPAF-C_n_ at 700–850 nm without the attachment of this type of antenna on the fullerene cage of **1**. Accordingly, we performed several femtosecond (fs) Z-scan measurements on four derivatives of **1**-C_n_ under the 2PA excitation at 780 nm in this study to verify the hypothesis, as a part of our effort to construct new nonlinear absorbing materials with broadband characteristics.

## 2. Results and Discussion

### 2.1. Materials Characterization

We have demonstrated the use of dialkyldiphenylaminofluorenyl-*keto*-[60]fullerene C_60_(>DPAF-C_n_) dyads **2**-C_n_ [16], branched triads C_60_(>DPAF-C_n_)_x_ (x = 2) [17], and the related starburst pentads (x = 4) [8] for the study of simultaneous 2PA phenomena under the photoexcitation of a 780-nm laser light in the fs region. In these compounds, DPAF-C_n_ was used as the light-harvesting antenna chromophore to compensate for the low optical absorption of the fullerene cage at wavelengths beyond 350 nm. It is also functioning as an electron donor to provide one electron capable of being transferred intramolecularly to the C_60_> cage moiety upon 2PA activation that increased largely the excited state absorptions leading to 2PA cross-section enhancement. As a general strategy to extend the active 2PA wavelength to the longer NIR region, we modified the bridging keto group in dyads **2**-C_n_ by an electron-withdrawing 1,1-dicyanoethylenyl (DCE) unit, leading to the structure of C_60_(>CPAF-C_n_) **1**-C_n_. The functional change resulted in the increase of molecular electronic polarization and bathochromic shift in the optical absorption from λ_max_ 400 nm for DPAF to 500–550 nm for CPAF. One example was given by C_60_(>CPAF-C_2M_) dyad [18].

**Scheme 1:**
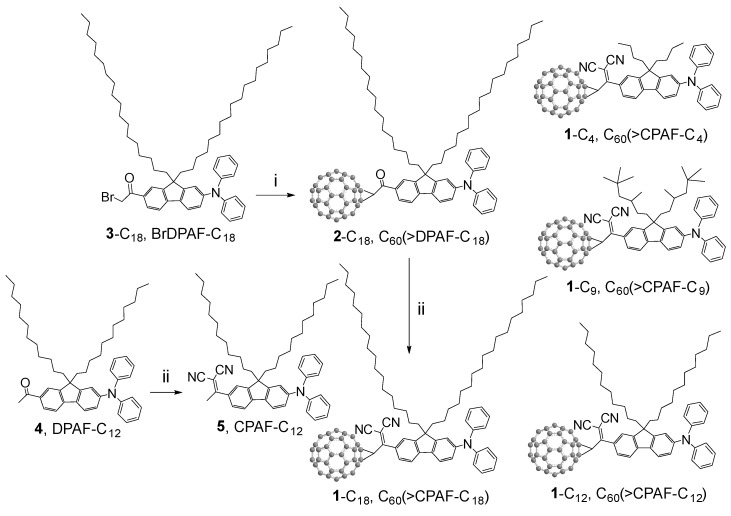
Synthesis of C_60_(>CPAF-C_n_) **1**-C_n_ (*n* = 4, 9, 12, or 18) dyads. *Reagents and conditions*: i. C_60_, DBU, toluene, rt, 5.0 h; ii, malononitrile, pyridine, TiCl_4_, rt, 5.0 min.

In the case of C_60_(>DPAF-C_9_)_x_ (x = 1, 2, and 4), their 2PA σ_2_ values were found to be concentration-dependent [8] with a higher magnitude at a lower concentration than 10^−3^ M. This revealed that a minimization of the molecular aggregation should be advantageous to prevent the loss of σ_2_ magnitude, especially at a high concentration for practical use. It is crucial since a sufficiently high 2PA material concentration may be required for the fabrication of NLO devices to bring in significant effects. Our logic approach is to modulate the compound’s solubility by the variation of attached alkyl chain length and shape (linearly or sterically hindered branched structures) and to control the effective average separation distance among C_60_> cages when applied in highly concentrated solutions or solid films. Accordingly, we synthesized four samples, namely, C_60_(>CPAF-C_n_) **1**-C_n_ (*n* = 4, 9, 12, and 18, Scheme 1) for the evaluation of their alkyl chain-dependent broadband 2PA characteristics. Since the 2PA activity of C_60_(>CPAF-C_n_) analogous moiety by the 980-nm excitation in toluene was reported recently using examples of C_60_(>CPAF-C_9_) and hybrid C_60_(>DPAF-C_18_) (>CPAF-C_2M_)_n_ (*n* = 1 or 2) [15], we investigated the σ_2_ value and the corresponding nonlinear absorption efficiency of the compound **1**-C_n_ under the excitation wavelength of 780 nm to substantiate their broadband two-photon absorbing properties.

**Figure 2:**
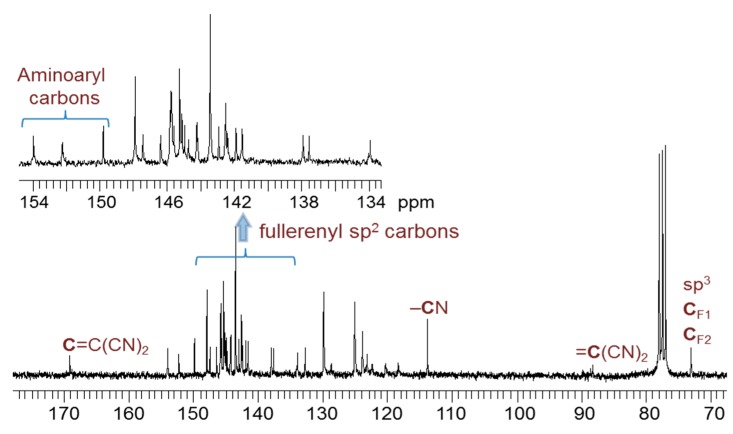
^13^C-NMR spectrum of C_60_(>CPAF-C_18_) showing all sp^2^ (δ 134–148) and two sp^3^ (C_F1_ and C_F2_) C_60_ cage carbons and 1,1-dicyanoethylenyl (DCE) carbons, as assigned.

**Figure 3:**
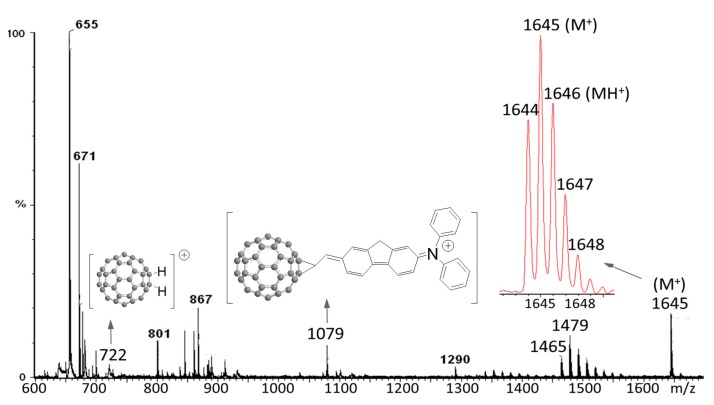
Matrix-assisted laser desorption ionization mass spectrum (MALDI-MS) of C_60_(>CPAF-C_18_) **1**-C_18_ using α-cyano-4-hydroxy-cinnamic acid as the matrix material, showing the molecular ion mass M^+^ (or MH^+^).

Synthesis of the compound C_60_(>CPAF-C_9_) **1**-C_9_ followed the procedure described previously [18]. A similar synthetic sequence was applied for the preparation of C_60_(>CPAF-C_4_) **1**-C_4_, C_60_(>CPAF-C_12_) **1**-C_12_, and C_60_(>CPAF-C_18_) **1**-C_18_. Formation of a fullerenyl monoadduct **1**-C_n_ was evident by detection of its infrared spectrum displaying three typical fullerenyl signals at 753, 697, and 527 cm^−1^ corresponding to absorptions of an unfunctionalized half-cage sphere of C_60_>. The key chemical modification of a keto group of C_60_(>DPAF-C_18_) **2**-C_18_ to a 1,1-dicyanoethylene (DCE) group of **1**-C_18_ was made by using malononitrile as a reagent. Indication of the CPAF moiety attached on a C_60_> cage was seen clearly by a strong IR absorption band corresponding to cyano (–C≡N) stretching vibrations centered at 2222–2224 cm^−1^ with complete disappearance of the carbonyl stretching vibration of **2**-C_18_ at 1680 cm^−1^. It was also substantiated by its ^13^C-NMR spectrum (Figure 2) giving chemical shifts of three types of functional carbons, –**C**=C(CN)_2_, –**C**≡N, and =**C**(CN)_2_, in the 1,1-dicyanoethylenyl moiety of **1**-C_18_ at δ 169.13, 113.84, and 88.22, respectively, confirming the successful conversion reaction. In Figure 2, it also showed chemical shifts of three aminoaryl carbons in CPAF-C_18_ moiety at δ 153.91, 152.18, and 149.82 along with all fullerenyl sp^2^ carbon peaks located within δ 134–148, whereas two sp^3^ C_60_> carbon (C_F1_ and C_F2_) peaks were assigned at δ 73.09. Direct confirmation of the molecular mass of **1**-C_18_ was made by a group of sharp molecular mass ions with the maximum mass intensity centered at *m*/*z* 1645 (M^+^) and 1646 (MH^+^) in its MALDI-TOF mass spectrum (Figure 3). This was followed by several groups of ion peaks at *m*/*z* 1465–1550 with the group mass each separated by a –CH_2_– unit (*m*/*z* 14) indicating the consecutive loss of alkyl chain carbons from the M^+^ peak. Full elimination of weaker aliphatic bonds of **1**-C_18_ led to a stable aromatic mass ion fragment at *m*/*z* 1079, matching with the structure assigned in the Figure. Further fragmentation gave stable C_60_^+^ (*m*/*z* 720) and C_60_H_2_^+^ (*m*/*z* 722) mass ion fragments.

**Figure 4:**
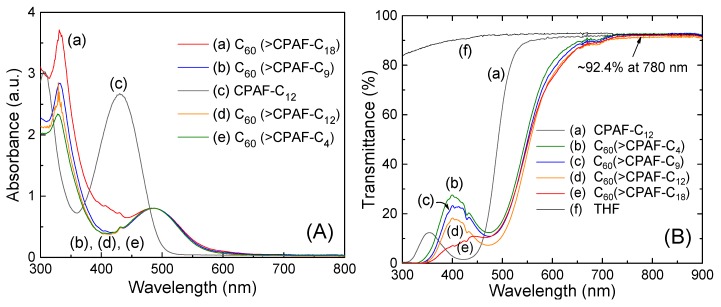
UV-vis (**A**) absorption (toluene, 1.0 × 10^−5^ M, normalized at λ_max_ 486 nm) and (**B**) transmittance (THF, 2.0 × 10^−5^ M) spectra of CPAF-C_12_, **1**-C_4_, **1**-C_9_, **1**-C_12_, and **1**-C_18_ with THF as the reference.

Similar to that of **1**-C_9_ [18], the keto modification of **2**-C_18_ led to a large bathochromic shift of the long-wavelength absorption band of **1**-C_18_ to λ_max_ 468 (ε = 4.2 × 10^4^ L/mol·cm, toluene, 1.0 × 10^−5^ M) or 503 nm (ε = 2.9 × 10^4^ L/mol·cm, CHCl_3_, 2.0 × 10^−5^ M) in nearly 58–93 nm longer than that of C_60_(>DPAF-C_18_) (**2**-C_18_) centered at λ_max_ 410 nm, as shown in Figure 4A-a. This band was accompanied with two other absorption bands with λ_max_ centered at 260 (ε = 1.7 × 10^5^) and 327 (ε = 8.2 × 10^4^) in CHCl_3_ or 326 (ε = 1.5 × 10^5^ L/mol·cm) in toluene, attributed to absorptions of C_60_ > cage. By comparing with the λ_max_ value of CPAF-C_12_ **5** [19] (Figure 4A-c) antenna alone at 437 nm, a longer absorption wavelength λ_max_ for all **1**-C_4_ (Figure 4A-e), **1**-C_9_ (Figure 4A-b), **1**-C_12_ (Figure 4A-d), and **1**-C_18_ (Figure 4A-a) giving dark burgundy-red in color, clearly revealing a partial conjugation between the CPAF moiety and a C_60_> cage, matching with our proposed endinitrile tautomeric resonance isomerization described in Figure 1. In addition, pronounced solvent-dependent optical absorption was detected that resulted in a longer wavelength in polar (CHCl_3_) than in non-polar (toluene) solvent. Similar solvent polarity-dependent photophysical properties were also observed for the analogous compound C_60_(>CPAF-C_2M_) exhibiting nanosecond transient intramolecular electron-transfer activity from the CPAF light-harvesting antenna to the C_60_> acceptor moiety in polar solvents (e.g., PhCN) upon photoexcitation while, in non-polar solvents such as toluene, intramolecular energy-transfer activity is the major event [19]. In the latter case, initial photoactivation at either C_60_> (300–350 nm for linear absorption or 600–700 nm for 2PA processes) to ^1^(C_60_>)* or CPAF-C_n_ (450–550 nm for 1PA or 900–1100 nm for 2PA) to ^1^(CPAF)*-C_n_ is followed by formation of the singlet ^1^C_60_*(>CPAF-C_n_) transient state in an ultrafast rate that was capable of crossing over to the triplet ^3^C_60_*(>CPAF-C_n_) transient state, via intersystem-crossing (ISC) in a time period of roughly 1.4 nanoseconds (ns) [17]. The triplet lifetime was 34–39 microseconds (µs) [18]. Therefore, in this study, using a pulse laser light operating at 226-fs for 2PA measurements carried out in THF or toluene, the singlet transient states ^1^C_60_*(>CPAF-C_n_) (*n* = 4, 9, 12, or 18) should be the main targets for the consideration of excited states and reverse saturable absorptions (RSA) [20] in correlation to the nonlinear absorption effect.  

### 2.2. Nonlinear Z-Scan Measurements

**Figure 5:**
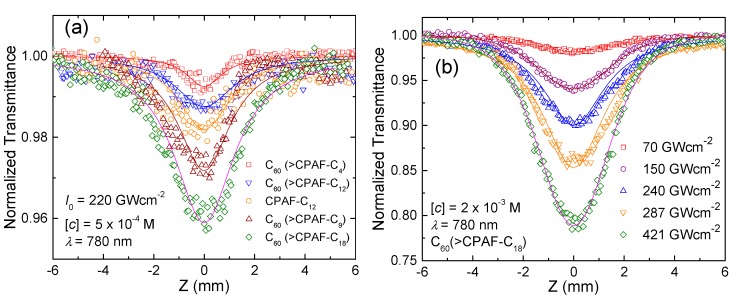
Open-aperture *Z*-scan curves of (**a**) CPAF-C_12_ and four C_60_(>CPAF-C_n_) monoadducts taken at 220 GWcm^−2^ in THF and (**b**) C_60_(>CPAF-C_18_) taken at different pulse laser intensities indicated.

The open-aperture Z-scans of four C_60_(>CPAF-C_n_) samples were carried out in THF with femtosecond laser pulses. *Z*-scan measurements carried out at an ultrafast time scale of 226 femtoseconds should be able to reduce potential accumulative thermal scattering effects, normally occurring at picosecond regions, at the wavelength of either 780 or 1000 nm. The transmittance of all compounds studied were collected, as shown in Figure 4B, indicating a consistent level of ~92.4% at 780 nm. The data reported in Figure 5a,b were normalized to the linear transmittance for all *Z*-scans by the correction of the background transmittance, *T*(|*Z*| >> *Z*_o_). The normalized transmittance ∆*T*(*Z*) was expressed as *T*(*Z*)/*T*(|*Z*| >> *Z*_o_). Accordingly, the change in the normalized transmittance is indicative of the nonlinear (or light-dependent) part in the compound’s absorption. Total absorption was described by the change in the absorption coefficient ∆α = β*I*, where β and *I* are the 2PA coefficient and the light intensity, respectively. The absorption coefficient can be extracted from the best fitting between the *Z*-scan theory [21] and the data. The 2PA cross-section value was then calculated from the coefficient by the formula σ_2_ = β*ħ*ω/*N*, where *ħ*ω is the photon energy and *N* is the number of the molecules.

Open-aperture *Z*-scans carried out under the irradiance of 220 GW/cm^2^ at 780 nm were taken on the samples of **5**, **1**-C_4_, **1**-C_9_, **1**-C_12_, and **1**-C_18_ in THF at the concentration of 5.0 × 10^−4^ M with the profile plots shown in Figure 5a. These *Z*-scans displayed positive signs for absorptive nonlinearities with the decrease of light-transmittance in the order of C_60_(>CPAF-C_18_) < C_60_(>CPAF-C_9_) ≤ C_60_(>CPAF-C_12_) < C_60_(>CPAF-C_4_) in solution. As a result, the 2PA cross-section values of these compounds measured were summarized in Table 1.

It is interesting to observe a higher 2PA absorption cross-section value of 6.42 × 10^−48^ cm^4^·s· photon^−1^·molecule^−1^ (or 642 GM) for C_60_(>CPAF-C_18_) at a low concentration of 5.0 × 10^−4^ M than that, 3.25 × 10^−48^ cm^4^·s·photon^−1^·molecule^−1^ (or 325 GM), for C_60_(>CPAF-C_9_) at the same concentration. A lower value of 1.28 × 10^−48^ cm^4^·s·photon^−1^·molecule^−1^ (or 128 GM) for C_60_(>CPAF-C_4_) than the un-fullerenized CPAF-C_12_ (220 GM) was detected, perhaps owing to its higher particle aggregation tendency even at 10^−4^ M. Based on a 226-fs pulse duration is slightly longer than 130 fs required for the intramolecular energy-transfer from the photoexcited ^1^(CPAF)*-C_n_ antenna moiety to the C_60_> cage of C_60_(>CPAF-C_n_). Completion of this energy-transfer event at the early time scale leads to the formation of excited ^1^C_60_*(>CPAF-C_n_) state. Therefore, the measured σ_2_ values at 226-fs should cover partly two-photon absorptions of both CPAF-C_n_ and C_60_> moieties in the fs region and the excited singlet state absorption (*S*_1_–*S*_n_) of ^1^(C_60_>)* cage moiety in subsequent subpicoseconds. The initial 2PA excitation process at 780 nm represents mainly the contribution of CPAF-C_n_ moiety forming the transient C_60_(>^1^CPAF*-C_n_) state. The argument is valid due to the fact of low linear and nonlinear C_60_> cage absorption at this wavelength as compared with the later of CPAF-C_n_ moiety. In addition, the occurrence of transient conversion from ^1^C_60_*(>DPAF-C_9_) state to the corresponding ^3^C_60_*(>DPAF-C_9_) state via inter-system crossing was reported to be effective at a much longer time scale of ~1.4 ns [17]. Therefore, the absorption contribution of ^3^C_60_*(>CPAF-C_9_) state can be excluded in this measurement. These nonlinear fs absorptions may be correlated to the following nonlinear absorption measurements.

**Figure 6:**
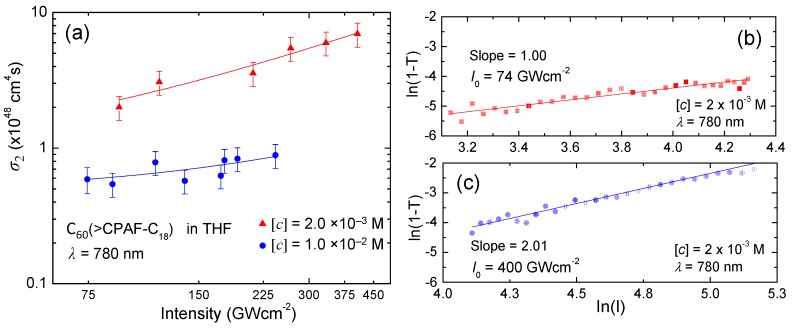
(**a**) Effective two photon absorption cross sections of C_60_(>CPAF-C_18_) plotted as a function of pulse laser intensities; (**b**) and (**c**) show the plot of ln(1‒T) *vs. I* for the same compound with different intensities *I*_o_ at the focal point.

**Figure 7:**
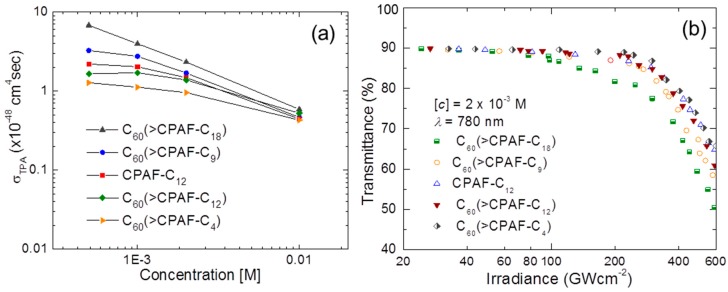
(**a**) Two-photon absorption cross-sections and (**b**) nonlinear absorption of **5**, **1**-C_4_, **1**-C_9_, **1**-C_12_, and **1**-C_18_ in THF (2.0 × 10^−3^ M) measured as a function of irradiance with 226-fs laser pulses operated at 780 nm.

We also investigated the intensity-dependent (70–420 GWcm^−2^) Z-scans using the compound **1**-C_18_ as an example in THF at a concentration of 2.0 × 10^−3^ M by 780-nm excitation. The resulting data profiles were displayed in Figure 5b with the corresponding 2PA cross-sections plotted in Figure 6a (red triangle). At this concentration, the σ_2_ values were higher, in general, and increased more rapidly than those taken at a higher concentration of 1.0 × 10^−2^ M (Figure 6a, blue circle) at the same laser intensity. This trend of concentration-dependent σ_2_ values having a higher quantity at a lower concentration consistent with that reported recently [8]. The intensity dependence on σ_2_ values may also reveal higher order absorptions, such as excited state absorption (ESA) that can be effectively treated as three-photon absorption (3PA) for ESA, possibly taken place. In order to distinguish the contribution of 2PA from the higher order nonlinear absorption, the ln(1–T) *vs.* intensity (*I*) relationship was plotted, as shown in Figure 6b,c. These *Z*-scan curves were fitted with 2PA when the slope is ~1.0 and fitted with ESA/3PA when the slope is ~2.0 [22]. The fitting results confirmed that, at a low laser intensity of 74 GWcm^−2^ (Figure 6b), the event of 2PA process dominates, while at a high intensity of 400 GWcm^−2^ (Figure 6c), the photophysical processes of ESA/3PA became the major occurrence. Accordingly, the ESA cross-sections (σ_ESA_) of **5**, **1**-C_4_, **1**-C_9_, **1**-C_12_, and **1**-C_18_ were determined and given in Table 1. They showed the similar trend of concentration dependence in magnitude to those of σ_2_ (Figure 7a) having a higher value at a lower concentration. The trend was also coupled with their solubility where **1**-C_18_ with two linear octadecyl chains and **1**-C_9_ with two branched 3,5,5-trimethylhexyl chains exhibit better solubility in solvents and a higher magnitude of σ_2_. We have examined several solvents including CS_2_, THF, and toluene using C_60_(>CPAF-C_18_) as the example under 780-nm excitation and found no significant difference in the value of σ_2_ indicating no solvent effect for the case.

Nonlinear absorption properties of C_60_>CPAF-C_n_) were investigated by irradiance-dependent transmission measurements at the wavelength of 780 nm using the same setup as those applied in 2PA cross-section measurements conducted by fs-laser pulses. Nonlinear absorption of CPAF-C_12_, C_60_(>CPAF-C_4_), C_60_ (>CPAF-C_9_), C_60_(>CPAF-C_12_), and C_60_(>CPAF-C_18_) in THF measured as a function of irradiance with 226-fs laser pulses operated at 780 nm were illustrated in Figure 7b. All the samples showed a linear transmission (*T* = ~90%) with input intensity of up to 30 GW/cm^2^. When the incident intensity was increased above 70 GW/cm^2^, the transmittance (%) began to deviate from the linear transmission line and decrease indicating the initiation of nonlinear absorption. A systematic trend showing higher nonlinear absorption efficiency down to 50%, 57%, 60%, and 65% for the dyads **1**-C_18_, **1**-C_9_, **1**-C_12_, and **1**-C_4_, respectively, was observed with the increase of irradiance intensity up to 600 GW/cm^2^ (Figure 7b). Improvement in lowering the transmittance can be correlated to the higher solubility of the dyads, consistent with the positive contribution of C_60_(>CPAF-C_18_) and C_60_(>CPAF-C_9_) to a larger transient absorptions, concluded by *Z*-scans in Figure 5a.

## 3. Experimental Section

### 3.1. Materials

Reagents and solvent of *n*-butanol, 3,5,5-trimethylhexanol, *n*-dodecanol, *n*-octadecanol, methanesulfonyl chloride, triethylamine, 2-bromofluorene, malononitrile, *rac*-2,2′- bis(diphenylphosphino)-1,1′-binaphthyl (*rac*-BINAP), tris(dibenzylideneacetone)dipalladium(0) [Pd_2_(dba)_3_(0)], aniline, and dichloroethane were purchased from Aldrich Chemicals (St. Louis, MO, USA) and used without further purification. C_60_ (99.5%) was purchased from NeoTech Product Co. (Moscow, Russia) and used as received. All other chemicals were purchased from Acros Ltd. (New Brunswick, NJ, USA). The anhydrous grade solvent of THF was refluxed over sodium and benzophenone overnight and distilled under reduced pressure (10^−1^ mmHg).

### 3.2. Spectroscopic Measurements

^1^H-NMR and ^13^C-NMR spectra were recorded on either a Bruker Avance Spectrospin–200 or Bruker AC-300 spectrometer (Bruker, Billerica, MA, USA). UV-vis spectra were recorded on a Hitachi U-3410 UV spectrometer (Hitachi, Chiyoda, Tokyo, Japan). Infrared spectra were recorded as KBr pellets on a Nicolet 750 series FT-IR spectrometer (Thermo Scientific Nicolet, Waltham, MA, USA). Mass spectroscopic measurements were performed by the use of positive ion matrix-assisted laser desorption ionization (MALDI-TOF) technique on a micromass M@LDI-LR mass spectrometer (Micromass, Cary, NC, USA). The matrix of 3,5-dimethoxy-4-hydroxycinnamic acid (sinapic acid) was used.

### 3.3. Synthetic Procedures

Synthesis of 7-(1,2-dihydro-1,2-methanofullerene[60]-61-{1,1-dicyanoethylene})-9,9-di(3,5,5- trimethylhexyl)-2-diphenylaminofluorene C_60_(>CPAF-C_9_), **1**-C_9_. Similar procedures as those reported [18] were used.

Synthesis of 7-α-bromoacetyl-9,9-dioctadecanyl-2-diphenylaminofluorene BrDPAF-C_18_ (**3**-C_18_). To a suspension of aluminum chloride (1.30 g, 9.66 mmol) in 1,2-dichloroethane (50 mL) at 0 °C was added a solution of 9,9-dioctadecyl-2-diphenylaminofluorene [23] (2.4 g, 2.9 mmol) in 1,2-dichloroethane (30 mL). It was then added by bromoacetyl bromide (0.56 g, 2.79 mmol) over 10 min. At the end of addition, the mixture was warmed to ambient temperature and stirred for an additional 15.0 h. The solution was diluted by a slow addition of water (100 mL) while maintaining the reaction mixture temperature below 45 °C. The resulting organic layer was washed subsequently with dilute hydrochloric acid (1.0 N, 50 mL) and water (2 × 50 mL), then, the solution was dried over magnesium sulfate and concentrated *in vacuo*. The crude yellow oil was purified by column chromatography (SiO_2_, hexane‒EtOAc, 9:1) to afford 7-α-bromoacetyl-9,9-di-(*n*-octadecyl)- 2-diphenylaminofluorene **3**-C_18_ (1.8 g, 78%) with its chromatographic band corresponding to *R*_f_ = 0.6 on TLC (SiO_2_, hexane‒EtOAc, 9:1 as the eluent); FT-IR (KBr) ν_max_ 3063 (w), 3034 (w), 2923 (s), 2852 (s), 1677 (m), 1595 (m), 1493 (m), 1466 (w), 1346 (w), 1279 (m), 1182 (w), 1027 (w), 819 (w), 753 (w), 697 (m), 620 (w), and 508 (w) cm^−1^; UV-vis (CHCl_3_, 1.0 × 10^−5^ M) λ_max_ (ε) 292 (1.9 × 10^4^) and 407 (2.5 × 10^4^ L/mol·cm); ^1^H-NMR (500 MHz, CDCl_3_, ppm) δ 7.95 (d, *J* = 8.18 Hz, 1H), 7.93 (s, 1H), 7.64 (d, *J* = 7.91 Hz, 1H), 7.59 (d, *J* = 8.23 Hz, 1H), 7.27–7.23 (m, 4H), 7.14–7.12 (m, 5H), 7.05–7.02 (m, 3H), 4.49 (s, 2H), 1.97–1.81 (m, 4H), 1.25–1.04 (m, 66H), 0.87 (t, *J* = 6.78 Hz, 6H), and 0.72–0.55 (br, 4H); ^13^C-NMR (126 MHz, CDCl_3_) δ 190.99, 153.63, 151.06, 148.81, 147.61, 146.89, 133.96, 131.55, 129.25, 128.80, 124.36, 123.09, 122.78, 121.61, 118.82, 118.20, 55.23, 39.96, 31.90, 31.15, 29.90, 29.67, 29.64, 29.62, 29.57, 29.55, 29.34, 29.29, 23.83, 22.67, and 14.10.

Synthesis of 7-(1,2-dihydro-1,2-methanofullerene[60]-61-carbonyl)-9,9-dioctadecanyl-2-diphenyl- aminofluorene, C_60_(>DPAF-C_18_), **2**-C_18_. To a mixture of C_60_ (0.75 g, 1.1 mmol) and 7-α-bromoacetyl-9,9-dioctadecanyl-2-diphenylaminofluorene **3**-C_18_ (0.85 g, 1.1 mmol) in dry toluene (500 mL) was added DBU (0.18 mL, 1.2 mmol) under a nitrogen atmosphere. After stirring at room temperature for 5.0 h, suspended solids of the reaction mixture were filtered off and the filtrate was concentrated to a volume of 10%. Crude products were precipitated by the addition of methanol and isolated by centrifugation (8000 rpm, 20 min). The isolated solid was a mixture of the monoadduct **2**-C_18_ and its bisadduct. Separation of these two products were done by column chromatography (silica gel) using a solvent mixture of hexane‒toluene (3:2) as the eluent. The first chromatographic band corresponding to *R*_f_ = 0.7 on TLC (SiO_2_, hexane‒toluene, 3:1) afforded C_60_(>DPAF-C_18_) **2**-C_18_, as brown solids (1.12 g, 65% based on recovered C_60_); FT-IR (KBr) ν_max_ 3440 (m), 2920 (s), 2849 (s), 1674 (m), 1632 (m), 1593 (s), 1491 (m), 1463 (m), 1427 (m), 1346 (w), 1331 (w), 1316 (w), 1273 (m), 1239 (w), 1200 (m), 1186 (w), 1157 (w), 1028 (w), 817 (w), 752 (m), 738 (w), 696 (m), 575 (w), 547 (w), 526 (m), and 490 (m) cm^−1^; UV-vis (CHCl_3_, 1.0 × 10^−5^ M) λ_max_ (ε) 260 (1.3 × 10^5^), 325 (4.7 × 10^4^), and 411 (3.6 × 10^4^ L/mol·cm); ^1^H-NMR (500 MHz, CDCl_3_, ppm) δ 8.43 (d, *J* = 6.9 Hz, 1H), 8.32 (s, 1H), 7.78 (d, *J* = 8.0 Hz, 1H), 7.61 (d, *J* = 8.0 Hz, 1H), 7.25–7.22 (m, 4H), 7.11–7.09 (m, 5H), 7.03–7.00 (m, 3H), 5.66 (s, 1H), 2.03–1.84 (m, 4H), 1.29–1.04 (m, 58H), 0.87 (t, *J* = 6.88 Hz , 6H), and 0.69 (br, 4H); ^13^C-NMR (126 MHz, CDCl_3_) δ 188.33, 153.55, 151.20, 148.77, 147.96, 147.30, 147.20, 146.73, 145.35, 145.24, 145.06, 144.96, 144.85, 144.70, 144.52, 144.43, 144.39, 144.13, 143.74, 143.49, 143.14, 142.96, 142.91, 142.83, 142.76, 142.57, 142.32, 142.07, 142.00, 141.90, 141.06, 140.76, 139.36, 136.46, 133.57, 133.22, 129.22, 128.62, 124.40, 123.15, 122.83, 122.42, 121.71, 119.14, 117.78, 72.48, 55.09, 44.58, 40.14, 32.00, 30.16, 29.81, 29.56, 29.47, 24.11, 22.87, and 14.22.

Synthesis of 7-(1,2-dihydro-1,2-methanofullerene[60]-61-{1,1-dicyanoethylene})-9,9-dioctadecanyl-2- diphenylaminofluorene C_60_(>CPAF-C_18_), **1**-C_18_. To a mixture of C_60_(>DPAF-C_18_) **2**-C_18_ (240 mg, 0.17 mmol) and malononitrile (29 mg, 0.34 mmol) in dry chloroform (30 mL) was added pyridine (52 mg, 0.68 mmol) with stirring under a nitrogen atmosphere. To this solution, titanium tetrachloride (0.20 mL, excess) was added in one portion. After stirring at room temperature for 5.0 min, the reaction mixture was quenched with water (30 mL). The resulting organic layer was washed several times with water (100 mL each), dried over magnesium sulfate, and concentrated *in vacuo* to afford the crude orange red solid product. It was purified by PTLC (SiO_2_, toluene‒hexane, 1:1). A product fraction collected at *R*_f_ = 0.8 (hexane‒toluene, 1:1) was identified to be C_60_(>CPAF-C_18_) **1**-C_18_ as orange-red solids in a yield of 50 mg (24%); MALDI-MS (TOF) calculated for ^12^C_126_^1^H_91_^14^N_3_ *m*/*z* 1647; found, *m*/*z* 655, 671, 722, 801, 867, 1079, 1290, 1465, 1479, 1647 (M^+^), 1648 (MH^+^); UV-vis (toluene, 1.0 × 10^−5^ M) λ_max_ (ε) 325 (1.5 × 10^5^), and 470 (4.3 × 10^4^ L/mol·cm or 260 (1.7 × 10^5^), 327 (8.2 × 10^4^), and 503 nm (2.9 × 10^4^ L/mol-cm) in CHCl_3_ (2.0 × 10^−5^ M); FT-IR (KBr) ν_max_ 2960 (w), 2923 (s), 2848 (m), 2222 (m), 1625 (s), 1596 (s), 1538 (w), 1489 (s), 1465 (w), 1345 (w), 1280 (m), 1265 (m), 1169 (m), 1089 (s), 1028 (m), 809 (w), 748 (w), 695 (w), 526 (s) cm^−1^; ^1^H-NMR (500 MHz, CDCl_3_, ppm) δ 8.12 (d, *J* = 8.12 Hz, 1H), 8.01 (s, 1H), 7.78 (d, *J* = 7.8 Hz, 1H), 7.59 (d, *J* = 7.9 Hz, 1H), 7.30–7.02 (m, 12H), 5.54 (s, 1H), 2.00–1.83 (m, 4H), and 1.40–0.71 (m, 70H); ^13^C-NMR (200 MHz, CDCl_3_, ppm) δ 169.13, 153.91, 152.18, 149.82, 147.92, 147.76, 146.45, 146.44, 145.74, 145.56, 145.51, 145.30, 145.18, 145.09, 144.81, 144.74, 144.22, 144.11, 143.53, 143.38, 143.32, 142.78, 142.51, 142.44, 141.78, 141.49, 137.89, 137.62, 134.00, 132.78, 129.90, 125.32, 123.82, 123.24, 123.13, 122.47, 120.02, 118.12, 113.84, 113.71, 88.22, 73.09, 50.49, 44.71, 33.92, 32.90, 31.98, 30.16, 29.82, 29.71, 29.56, 29.37, 29.31, 26.13, 25.58, 22.72, 21.53, 19.52, 17.21, and 14.07.

Synthesis of 7-(1,2-dihydro-1,2-methanofullerene[60]-61-{1,1-dicyanoethylene})-9,9-dibutyl-2- diphenylaminofluorene C_60_(>CPAF-C_4_), **1**-C_4_. Similar procedures as described above for **2**-C_18_ and **1**-C_18_ were applied to obtain C_60_(>CPAF-C_4_) as orange-red solids in a yield of 28%; MALDI-MS (TOF) calculated for ^12^C_98_^1^H_35_^14^N_3_ *m*/*z* 1254; found, *m*/*z* 1254 (M^+^), 1255 (MH^+^); UV-vis (toluene, 1.0 × 10^−^^5^ M) λ_max_ (ε) 323 (1.5 × 10^5^), and 485 (4.0 × 10^4^ L/mol-cm); FT-IR (KBr) ν_max_ 3027 (w), 2954 (m), 2925 (s), 2854 (m), 2224 (m), 1594 (vs), 1491 (m), 1281 (s), 1096 (s), 819 (m), 754 (s), 577 (w), 527 (m) cm^−1^; ^1^H-NMR (500 MHz, CDCl_3_, ppm) δ 8.15 (d, *J* = 8.2 Hz, 1H), 8.05 (s, 1H), 7.82 (d, *J* = 7.6 Hz, 1H), 7.62 (d, *J* = 8.2 Hz, 1H), 7.34–7.05 (m, 12H), 5.58 (s, 1H), 2.03–1.88 (m, 4H), and 1.08–0.63 (m, 14H).

Synthesis of 7-(1,2-dihydro-1,2-methanofullerene[60]-61-{1,1-dicyanoethylene})-9,9-didodecanyl-2- diphenylaminofluorene C_60_(>CPAF-C_12_), **1**-C_12_. Similar procedures as described above for **2**-C_18_ and **1**-C_18_ were applied to obtain **1**-C_12_ as orange-red solids in a yield of 24%; MALDI-MS (TOF) calculated for ^12^C_114_^1^H_67_^14^N_3_ *m*/*z* 1479; found, *m*/*z* 1479 (M^+^), 1480 (MH^+^); UV-vis (toluene, 1.0 × 10^−^^5^ M) λ_max_ (ε) 326 (1.5 × 10^5^), and 468 (4.2 × 10^4^ L/mol·cm); FT-IR (KBr) ν_max_ 3064 (w), 3036 (w), 2954 (m), 2923 (s), 2851 (w), 2224 (m), 1594 (vs), 1538 (w), 1492 (s), 1279 (s), 1186 (m), 1105 (vs), 820 (w), 753 (m), 697 (m), 527 (s) cm^−1^; ^1^H-NMR (200 MHz, CDCl_3_, ppm) δ 8.17 (d, *J* = 8.0 Hz, 1H), 8.06 (s, Hz, 1H), 7.83 (d, *J* = 8.0 Hz, 1H), 7.64 (d, *J* = 8.0 Hz, 1H), 7.40–7.06 (m, 12H), 5.59 (s, 1H), 2.4–1.7 (m, 4H), and 1.50–0.71 (m, 46H).

### 3.4. Z-scan and Light-Intensity Transmittance Measurements

*Z*-scan measurements. Open aperture Z-scan and the nonlinear transmittance experiments were carried out with femtosecond laser pulses at 780 nm. The full width at half maximum (FWHM) of the laser pulses was 226 ± 10 fs with the repetition rate of 1.0 kHz. In general, a sample of the compound was dissolved in various solvents (THF, toluene, or CS_2_) with four concentrations from 10^−4^ to 10^−2^ M studied and kept in 1.0-mm-thick quartz cuvette. The beam waist at the focal point was 18 ± 2 µm which corresponded to 1.0–1.6 mm diffraction length. Laser pulses were generated by a mode-locked Ti:Sapphire laser (Quantronix, IMRA America, Inc., Detroit, MI, USA), which was seeded by a Ti:Sapphire regenerative amplifier (Quantronix‒Titan, Marlborough, MA, USA), and was focused onto a 1.0-mm-thick quartz cuvette containing a solution of C_60_(>CPAF-C_n_). Incident and transmitted laser intensities were monitored as the cuvette being moved (or *Z*-scanned) along the propagation direction of laser pulses.

Light-intensity transmittance measurements. Similar experimental set-up and conditions as those of open aperture Z-scan measurements were applied for the nonlinear transmittance experiments at 780 nm. All compounds were dissolved in THF in the concentration of 2.0 × 10^−3^ M and kept in 1.0-mm-thick quartz cuvette. The transmittance data were collected upon the variation of irradiance intensity from 20 to 600 GWcm^−2^.

## 4. Conclusions

Nonlinearity and the light-intensity transmittance reduction effect of four C_60_(>CPAF-C_n_) (*n* = 4, 9, 12, or 18) monoadducts was substantiated by the 226-fs irradiance-dependent measurements above the incident irradiance of 70 GW/cm^2^ at 780 nm. A systematic trend showing higher efficiency in nonlinear absorption by the dyad C_60_(>CPAF-C_18_) than that of the other dyads was correlated to its higher multiphoton absorption (MPA), including 2PA and 3PA/ESA, cross-sections. We suggested the attachment of linear long-alkyl or branched alkyl chains being beneficial to the enhancement of 2PA and excited-state absorption owing to the minimization of molecular aggregation, including dimerization-induced self-quenching effect. In our analyses, excited-state absorption (ESA) is effectively treated as three-photon absorption (3PA). A clear concentration-dependent MPA cross-sections (σ_2_ and σ_ESA_) magnitude was detected showing a higher value at a lower concentration that was correlated to an increasing molecular separation with less aggregation in solution.

By taking the σ_2_ values of C_60_(>DPAF-C_9_) obtained at 780 nm photoexcitation in a 160-fs time scale previously [17] for comparison, we were able to explain a smaller fs σ_2_ 2PA cross-sections of C_60_(>CPAF-C_n_) using the same excitation wavelength being due to its lower linear absorption coefficient at 400 nm than that of C_60_(>DPAF-C_9_). Since the design of compounds **1**-C_n_ was aimed to extend the 2PA wavelength up to 1100 nm from that of C_60_(>DPAF-C_n_) analogous at 800 nm, their capability to function as 2PA and ESA/3PA absorbers even at 780 nm made them effective broadband NLO materials. This led to the corresponding light-intensity transmittance reduction efficiency. We suggest that the observed broadband absorptions may be attributed to a partial π-conjugation between the C_60_> cage and CPAF-C_n_ moieties, via endinitrile tautomeric resonances, involving a fully conjugated transient resonance state, as depicted in Figure 1. This conjugation form may enhance 2PA absorptions of **1**-C_n_ at 780 nm.

## Figures and Tables

**Table 1:** Two-photon absorption cross sections (σ_2_) and excited state absorption (ESA) cross-sections (σ*_ESA_*) of CPAF-C_12_ and C_60_(>CPAF-C_n_) measured using laser pulses working at 780 nm with a 226-fs duration and a repetition rate of 1.0 kHz. The light intensity *I* was 220 GW/cm^2^.

Sample	[C]/M	β/cmGW^−1^	σ_2_/10^−48^ cm^4^·s·photon^−1^·molecule^−1^	σ_ESA_/10^−78^ cm^6^·s^2^· photon^−2^·molecule^−1^
CPAF-C_12_	5.0 × 10^−4^	0.0026	2.20 (220 GM)	
	1.0 × 10^−3^	0.0048	2.03 (203 GM)	
	2.0 × 10^−3^	0.0070	1.48 (148 GM)	6.4
	1.0 × 10^−2^	0.0105	0.44 (44 GM)	1.5
C_60_(>CPAF-C_4_)	5.0 × 10^−4^	0.0015	1.28 (128 GM)	
	1.0 × 10^−3^	0.0027	1.12 (112 GM)	
	2.0 × 10^−3^	0.0045	0.95 (95 GM)	5.9
	1.0 × 10^−2^	0.0102	0.43 (43 GM)	
C_60_(>CPAF-C_9_)	5.0 × 10^−4^	0.0039	3.25 (325 GM)	
	1.0 × 10^−3^	0.0065	2.75 (275 GM)	
	2.0 × 10^−3^	0.0080	1.69 (169 GM)	10.2
	1.0 × 10^−2^	0.0110	0.46 (46 GM)	5.4
C_60_(>CPAF-C_12_)	5.0 × 10^−4^	0.0020	1.65 (165 GM)	
	1.0 × 10^−3^	0.0040	1.71 (171 GM)	
	2.0 × 10^−3^	0.0065	1.39 (139 GM)	9.1
	1.0 × 10^−2^	0.0124	0.535 (54 GM)	4.4
C_60_(>CPAF-C_18_)	5.0 × 10^−4^	0.0077	6.42 (642 GM)	30.1
	1.0 × 10^−3^	0.0094	3.97 (397 GM)	24.7
	2.0 × 10^−3^	0.0105	2.14 (214 GM)	11.3
	1.0 × 10^−2^	0.0139	0.59 (59 GM)	5.1
